# Experiences of fasting during Ramadan in British Muslims: Psychological, social and health behaviours

**DOI:** 10.1371/journal.pone.0313688

**Published:** 2025-01-09

**Authors:** Aaminah Latif, Syka Iqbal, Eleanor J. Bryant, Valerie E. Lesk, Barbara J. Stewart-Knox

**Affiliations:** Department of Psychology, University of Bradford, Bradford, United Kingdom; Lahore Medical and Dental College, PAKISTAN

## Abstract

Ramadan is a month-long religious festival observed by Muslim worldwide, characterised by intermittent fasting. This qualitative study addressed the need to understand how fasting is experienced by Muslims residing in Western cultures, aiming to inform policies that create a more supportive environment. Practicing Muslims, both men and women, were recruited in the North of England in the United Kingdom (UK). Data were collected by individual interviews (N = 7) and focus group discussion (N = 4) and then analysed using Social Ecological Theory as a framework. Fasting was managed at the individual level through instrumental food choice and eating practices, and by adapting sleep routines. Disrupted sleep routines posed a challenge for those who had to adhere to Western working schedules, leading to perceived detriments to cognitive function and mood. The sense of belonging associated with Ramadan was seen as a motivating factor for fasting. Breaking the fast (Iftar) was marked by social activity and the availability of traditional fried foods. Participants identified the wider Western culture and environment as challenging for those who are fasting. These findings imply a need for policies that enable flexible working practices for Muslims during Ramadan.

## Introduction

Ramadan is the Muslim month of fasting which occurs during the 9^th^ Islamic month of a lunar calendar. During this time, Muslims abstain from food and fluids (including water) from dawn until sunset. In equatorial countries such as Kenya and Somalia, fasting can last up to 15 hours, while in northern countries such as Iceland, fasting can last for up to 21 hours during the summer months. This variation in latitude and daylight time can significantly impact how individuals function during Ramadan. In the UK, where daylight hours are long in spring and summer, Muslims experience fasting periods of over 16 hours. With 6.5% of the United Kingdom (UK) population identifying as Muslim, making Islam the second-largest religion in the country, understanding the challenges they face while observing Ramadan in a Western context is essential [1]. Whilst UK Muslims have been fasting throughout the month of Ramadan for many years, there is a lack of research investigating how they navigate these challenges.

Quantitative studies that have investigated the cognitive performance during Ramadan have produced mixed results with some finding a decline in function [2, 3] and others indicating no change [4–6]. Quantitative studies of mood while fasting during Ramadan appear more consistent with some evidence for enhanced positive mood [4, 6–8] and reduced negative mood [4, 6]. However, such studies need to be interpreted with caution, given the small samples employed [4–7].

Together, this implies a need for in-depth qualitative research to capture peoples’ experiences of fasting and psychological functioning during Ramadan. Most existing qualitative studies of the psychology of Ramadan fasting have focussed upon clinical patient groups [2, 9–13] with only a few exploring psychological factors in Muslims practising Ramadan in Western cultures [14–16]. One study suggests that Ramadan fasting in a predominately Western culture can be associated with anxiety around eating and body image, while other qualitative studies in non-clinical groups indicate perceived benefits for mood and psychological wellbeing [17–19]. Notably, no existing qualitative studies have examined perceived cognitive performance during Ramadan fasting. The few studies in non-patient groups imply that it enhances self-efficacy, enabling people to eat more healthily [15, 19]. The little evidence on food choice during Ramadan suggests that although the calorific content remains similar, the proportion of carbohydrates and protein increases while fat decreases [20]. The reasons for such changes remain unclear.

Social Ecological Theory (SET) examines the interplay between physical, social and environmental factors upon a person’s psychological wellbeing and their health behaviour and holds that the greater the congruence/compatibility between the individual and the physical, social cand environmental context in which they function, the better their well-being [21]. For Muslims in Western societies, a lack of congruence with their cultural and philosophical backgrounds may impact their well-being. SET has been used to qualitatively analyse food choice and eating behaviour in minority groups resident within various global regions [22–25]. This analysis has employed SET to understand the challenges encountered in observing fasting during Ramadan and serves as a first step in designing intervention to raise awareness around nutrition during Ramadan and to improve health and wellbeing among Muslims in the UK.

Existing research highlights the importance of social factors in eating behaviour [26–28] and suggests perceived social benefits of Ramadan fasting [11, 14–16, 18, 19, 29, 30]. Self-Determination Theory (SDT) seeks to explain how social factors interact with motivation in determining wellbeing within the social environment [31]. According to SDT, people are more self-determined and motivated when they feel *competent* to master a behaviour, when they experience *relatedness* to others and have *autonomy* to choose to act [31]. SDT has been employed to analyse quantitative data on motivation and eating behaviour in different countries and social contexts [32, 33] and to interpret qualitative data on dietary health topics [28]. In this study, SDT serves as a framework for understanding the influence of social factors in motivation to fast during Ramadan.

It is important to understand experiences of Ramadan in Muslims residing in Western cultures such as that in the UK, and how this is perceived to impact health, food choice, social and psychological wellbeing and functioning, so that they can be better supported in observing the practice of fasting and in functioning effectively. Fasting may be made more difficult where societal practices are not adapted to take Ramadan into account. The aim of this research therefore has been to build theory to inform intervention and policy to accommodate Ramadan in the UK. Among the objectives will be to determine what (if any) adjustments are needed to societal institutions (e.g. workplaces, education establishments) and other environments to accommodate Ramadan so that those who are fasting during Ramadan are not disadvantaged.

## Methods

### Ethical approval

Approval for the study was granted by the University of Bradford Humanities, Social and Health Sciences Research Ethics Panel in January 2018. Title: Perspectives on British Ramadan; Ethics Application Ref: E655.

### Design and setting

A qualitative approach was taken using semi-structured interviews and a focus group discussion to provide a rich and detailed account of the experiences of Ramadan practices of British Muslims. Sampling and data collection were conducted in Bradford, which is a city located in the North of England, that has a multi-ethnic population in excess of 500,000 people, of whom 30.5% in the district and 60% in the city identified as Muslim in the 2021 Census [34].

### Sampling

A purposive sampling approach was employed to obtain a range of perspectives on Ramadan. For the interviews, we sought to recruit men and women of a range of adult age and employment. A focus group was held in addition to the interviews to capture the group dynamic associated with Ramadan and to establish data saturation. All focus group volunteers were women to capture women’s unique experience of fasting. Participants in the focus group were younger women, who were students. This allowed us to examine whether similar themes arose in a younger cohort and to explore how academic priorities influenced their fasting experiences. The inclusion of this group helped us confirm (or otherwise) whether the findings that emerged in focus groups held in this younger population, while also aiming for a holistic understanding of Ramadan fasting across life stages.

Inclusion criteria were being Muslim and having practiced fasting during Ramadan. Exclusion criteria were being non-Muslim, under the age of 18 years, having been diagnosed with a mental health condition, an eating disorder, a dietary health condition, a chronic or terminal illness or being pregnant or breastfeeding. All participants identified as Muslim, had lived in the UK for at least two years and had fasted during the 2019 month of Ramadan.

### Data collection

One to one interview and focus group discussions were chosen as suitable data collection tools for the purpose of this study. An interview guide was developed by the research team using open-ended questions to facilitate discussion. The start and end date of the recruitment period for this study occurred from 01/02/2019 until 29/04/2019. In addition to the seven interviews analysed, three pilot interviews were conducted to determine the design and content of the final topic guides for both the interview and focus group discussion (see Appendices 1 and 2 in S1 File). These pilot interviews were not included in the final analysis, The interview topic guide was adapted to tailor the questions for the focus group discussion and to obtain consensus on themes arising from the interviews. Participants were asked to sign a consent form and to provide demographic information. Focus group participants signed a confidentiality agreement to not communicate any information disclosed during discussion. Interviews and the focus group discussion took place within a private room on the University campus. Data collection concluded after saturation was reached following the completion of both the interviews and the focus group discussion. While initial themes were identified through the interviews, the focus group confirmed that no further themes emerged, thereby supporting data saturation.

### Reflexivity note

It is important to discuss the authors’ positionality within the research study to ensure transparency and credibility of the research process. The researcher who conducted the interviews and moderated the focus group discussion held insider status (AL), was bi-lingual in English and Urdu and a British Muslim woman of Pakistani heritage who resided in a town peripheral to Bradford. One of the data analysts was of British Indian heritage and a practising Muslim with a special interest in health inequalities. Both the researcher and data analyst, being practising Muslims who fasted during Ramadan and shared a similar cultural and religious background with the participants, likely influenced the sampling and enriched the quality of the data collected. The other data analyst (BS-K) was a woman of Irish nationality with no religious belief and a special interest in food psychology. Our similar and different positions and knowledge that we brought to the research allowed us to question and verify each other’s interpretation of the data.

### Data analysis

Individual interviews and the focus group discussion were audio recorded, transcribed verbatim and anonymised. Data were analysed using reflexive thematic analysis [35]. There are four essential steps in thematic analysis: familiarisation; initial thoughts, categories and themes; core themes; and reviewing themes [36]. Themes were then organised using a deductive (theory-driven) approach and agreed upon by all three researchers. The stages of analysis to establish initial coding were aided by ‘memo writing,’ by which reflections on the interview, prompted and unprompted themes, points of tension were recorded. This coding and memo-writing process aided in the subsequent thematic analysis. Thematic analysis is considered a foundation method for qualitative data analysis and is flexible enough to be incorporated into any epistemological approach [36]. The thematic analysis was conducted by three researchers (AL; BS-K; SI) who initially took an inductive (bottom-up/data driven) approach to generating themes for ensuring credibility and trustworthiness in the findings. Once the themes were generated, they were then organised according to the three pillars of SET (individual; social; environment) for the purpose of interpretation. This method allowed the broad SET domains to structure the themes, while the inductive coding informed the sub-themes within each domain.

## Results

### Sample description

The interviewees (N = 7) comprised four men aged 26–47 years and three women aged 22–46 years. Interviewees were Muslims who identified as British Asian (n = 3), Pakistani (n = 2), Arab (n = 1) and Bengali (n = 1). Interviews were semi-structured and lasted approximately 30–60 minutes. The focus group discussion participants were women (n = 4) aged 18–19 years, all of whom were students and who identified as British Pakistani. Although the participants were known to each other as classmates through the research participant information system, they were not part of a pre-existing friendship group.

### Main themes

**Fig 1:**
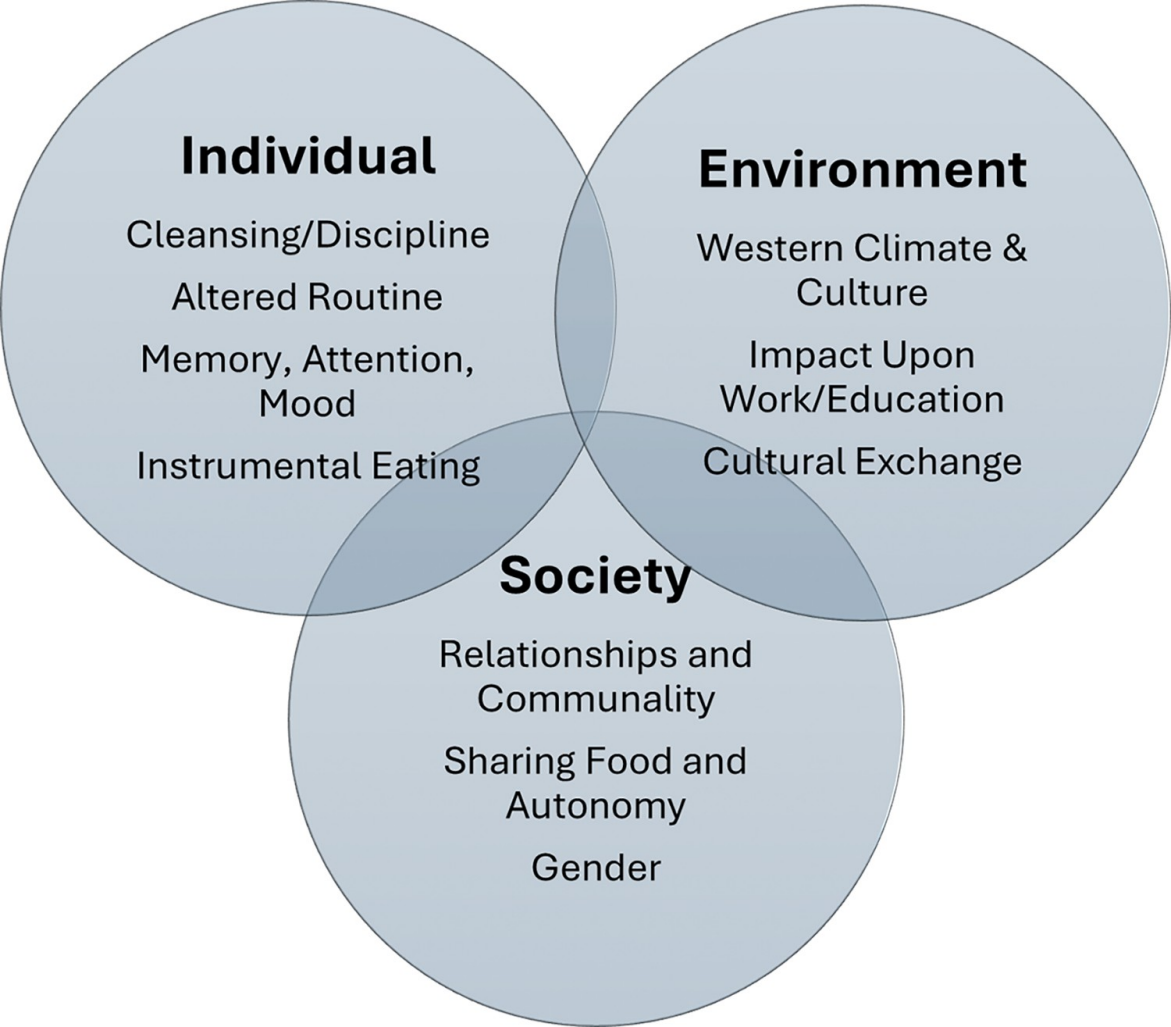
Experiences of fasting during Ramadan showing main individual, social and environmental themes and sub-themes.

Following inductive analysis, a deductive approach was taken to map the themes to reflect the experience of Ramadan fasting using Social Ecological Theory (SET) [21] as a framework (Fig 1). The constructs of the SET represent domains experienced at the individual level (Table 1), fasting in the social context (Table 2) and the intersection of fasting with the wider environment (Table 3). Food and eating formed a major theme that cut across all four major domains. Whilst individuals were concerned with managing fasting in daily life, Iftar (breaking the fast) heralded the social side of Ramadan which was around the collective culture of feasting. Experiences at the environmental level were concerned with fasting alongside a Western culture. Main themes were organised into the three broad categories reflecting the SET ‘individual’, ‘social’ and ‘environmental’ domains, each containing core themes and sub-themes.

**Table 1:** Individual themes.

Theme	Sub-Theme	Social Ecological Theory and Exemplary Data
**Meaning of Ramadan**	A detox—cleansing	“it’s er, it’s a physical and mental and spiritual erm cleansing process which enables us to basically become healthier, happier and stronger” (P1) “so it’s always like that time to recharge [laughs] erm throughout the year” (P5) “it’s just like literally a **detox** for your body and spiritual detox” (P6)
	Commitment Discipline	“when you’re fasting, you’re **capable** of doing anything, it’s all about putting your mind to” (P1) “it’s a discipline and then you know that when you’re fasting it’s almost like erm **you’ve made a pledge** and you don’t want to break that pledge” (P7)
**Physical Impact of Fasting**	Fasting physically demanding	“you know you go to the mosque for an evening prayer, which lasts for about an hour, and that is quite a physical, physically **demanding** kind of activity because you’re on your feet, standing and moving and you’re going through the motions of prayer” (P?) “There comes a point where you start getting aches and pains in your body, you start getting headaches, **you can’t then do your job**” (P3)
	Physical Fatigue	“I was just doing much lesser but **dragging myself** to go places. Dragging myself to get ready’ (P1) “in Ramadan, you do get tired …I suppose it was a bit of a, **bit of a drag** to sort of get yourself out into the gym and then train” (P2)
	Labile blood sugar	“Your **sugar levels** have really dropped, and that is the time when you just want peace and maybe want to lie down and go to sleep” (P1) “you’re eating more carbs and it’s spiking your blood sugars and your **blood sugars** are dropping” (P2)
**Disruption to Routines**	Living nocturnally	“my **sleeping pattern changes**. So, usually I probably go to sleep at 11 or 12 o’clock at night… in Ramadan what I’ll try and do, I wouldn’t go to sleep until after the dawn prayer. So after the Fajr prayer, maybe about 4 o’clock I’d go to sleep” (P1) “a normal day I probably sleep, 6, 6 and a half hours, 7 hours. During Ramadan it’s **probably 3 hours a night**, so” (P3) “I become a bit **nocturnal** in Ramadan… it was a bit more of a like night-time thing, a night-time routine rather than a day-time” (P6)
	Eating patterns	“… because you know **you’re eating at such strange times’** (P3) **“**Then like I stay up till like Suhoor time so like have something to eat before we have to start fasting and then yeah and then go to sleep after that**”** (P6)
**Psychological** **Impact of Fasting**	Detriment to memory and attention Exam performance	“I could still revise (for exams) but it would **take me a few times**” (P1) “it’d be a little bit **difficult er to fast and to do exams** as well and to concentrate if you don’t know how fasting affects your body, but if you understand how it affects your body” (P2) “so when I actually did do my tests and stuff I didn’t fast on the day, because when I did try I couldn’t concentrate. Its like **my recall and everything is completely gone**. I can’t remember anything’ (P3) “I think I’d feel more awake I guess and not, like at the end of an exam **I was just tired**, like I just wanted to sleep whereas on a normal exam I’d be fine really’ (P4) “If she has studied well and she has had enough sleep and everything it shouldn’t really and I feel it shouldn’t but the majority might think different because the **concentration**” (P7)
	Negative mood	‘I was very moody. So if someone spoke, speaking to me I’ll just like, I would look at them …’ (P3) ‘I remember being very **angry**” (P7)
**Determinants of Food Choice** **Instrumental Eating** **Eating Light**	Eating to improve performance and reduce fatigue	“Er and they’ll also give you the energy to get through what is gonna possibly a busy period, in the 4 hours when you’re gonna be going praying the night prayers as well” (P1) “when you’re eating healthily, you’re **not as tired and you can do more**” (P5) “….. well, you try to, you make healthy choices because you want to erm **preserve your energy** and everything” (P7)
	Eating to combat fatigue and prevent stomach pain	“always try to make sure that it’s healthy and it’s not gonna be **heavy** …. I mean what I try to do is, during the Iftar just stick to fruits and fluids, erm and then maybe a grilled, a grilled meat or kind of savouries” …“and also smaller portions because the smaller you eat, erm the less hunger pains and **less kind of drain** that you feel when you fasting” (P1) “so you’ve got the Taraweeh prayer which is an hour and a half Esha time so, the fact of the matter is, if you’re eating heavy how are you praying that prayer, because it’s literally difficult to stand up if you’re eating heavy” (P3) “when you’ve not eaten for so long and then you shock your system with like something really **heavy**, I feel like it would give me a stomach ache” (P6)
	Choosing Fruit	“I could just open my fast with like fruits and then after I would find it difficult to eat more than fruit” (P1) “No because I think when you open your fast you have your set **routine**. You open it, you have your date, have your date, you have water. By the time you’ve drunk your water you’re sort of half full anyway.” (P5) “I try to eat clean, more fruits during Ramadan. Erm pretty much more and more fruits that stuff” (P4)

**Table 2:** Social themes.

Theme	Sub-Theme	Social Ecological Theory and Exemplary Data
**Ramadan a Social Activity**	Renewal of relationships	“you can improve yourself as an individual and erm you know as an individual and a collective” (P1) “I usually have a, a thing where at the start of the month I sort of communicate with the people that I’m close to, friends and family, colleagues. Erm and I say to them, you know I’m starting the month of fasting, you know forgive me for any upset that I have caused you, so that there’s sort of a burden, the relief, that you know you started the month afresh, you sort of you’re wiping all of the things, your sorrows, you you know the things you may have said and done to people. You’re wiping the slate clean basically, you’re starting afresh” (P3)
	*Relatedness*	“Er, secondly there’s obviously the cultural value of fasting around family, around friends, particular time of year. When you’re up in the morning to close your fast you know that everybody in the street is awake” (P3) “Then there’s like the, there’s also like the social aspect of Ramadan coz like family’s come together a lot more in Ramadan. You meet a lot more friends for Iftar and stuff like that so, yeah er” (P5) “I think when you fast in Ramadan like everyone’s doing it sort of thing, it’s a group thing isn’t it really” (FgP4)
	Less talking during the fast	“I, tend to kind of reduce my, erm [pause], interactions with people, simply because of I just **talk less** during Ramadan. Erm, so that’s something that, that is impacted” (P1) “you tend to talk less because you don’t really have a lot of energy to speak to all erm the guys around the workplace. So, what I tend to do is I don’t really speak to anyone. Until or unless it’s very important and there’s something I need to speak to if not, **I don’t really speak**” (P4)
	*Competence*	‘I think for me like, they could always make up their fast afterwards. But then again, **it’s hard when you’re not doing it with everyone**, you’re doing it on your own, which is a lot worse’ (P3)
**Social** **Determinants of Food Choice**	Family *Autonomy*	‘sort of erm wary of **family** bakes that you have to eat that really coz I can’t be bothered making my own stuff” (P2) “I tend to have like a curry once a week or twice a week if my **mums** really forced it down my throat” (P3)
	Eating heavy *Relatedness* *Autonomy*	“it’s easy to actually eat, to eat worse because you’re eating a lot of fried food and you’re eating a lot of fatty foods, maybe they’re in the morning meals and then **fatty and fried foods** for the Iftar” (P1) “I just eat in the evening but then you’re eating whatever er is, is there. So it could be like erm a lot of **fried food**” (P2) “you’re starving all day and then all of a sudden you’re **eating so much**. It doesn’t make sense that doesn’t give you the sense of Ramadan either” (P4) “I didn’t eat any fried food at all but when I did go to my friend’s house, if they’ve had fried food then I would eat it” (P7)
**Engaging with Wider Society on Ramadan**	Perceived awareness and readiness among wider society to engage	“**people are quite interested** and want to know more and they’re fascinated”… “Ramadan is a fantastic opportunity to engage with people of other faiths and bring them into you know” (P1) “it’s becoming more and more, more and more erm, there’s more and **more awareness** of it in like health, from a health perspective” (P2)
	Fasting as an obligation *Autonomy*	“it’s also obligatory. Er, so as a practising Muslim, er you know this is an **obligation**, so it’s **not just a kind of voluntary thing** that you’re doing, it’s actually an obligation” (P1) “If there was a better understanding of, oh, they have to do this, they **don’t really have a choice** then they might be a little bit more accommodating” (P6)
	Working with non-Muslim colleagues	“employers may see there might be some positives in this for them, they might be more, employees might be more productive, erm so there’s actually, they could be some tangible benefits for them as well as employees” (P1) “they were changing a few things …that nurse who were fasting could have a break, to break their fast and it wasn’t very welcomed by other staff” (P7)
	Need to communicate	“Nobody’s tackled the problems, nobody has decided that we need to teach our youth what are the ways of life in modern Britain” (P3) “there’s a lot of things that going on around about Muslims and everything. So **they think we Muslims are nutters and they shouldn’t be doing this**” (P7)

**Table 3:** Environmental themes.

Theme	Sub-Theme	Social Ecological Theory and Exemplary Data
**Ramadan a Social Activity**	Renewal of relationships	“you can improve yourself as an individual and erm you know as an individual and a collective” (P1) “I usually have a, a thing where at the start of the month I sort of communicate with the people that I’m close to, friends and family, colleagues. Erm and I say to them, you know I’m starting the month of fasting, you know forgive me for any upset that I have caused you, so that there’s sort of a burden, the relief, that you know you started the month afresh, you sort of you’re wiping all of the things, your sorrows, you you know the things you may have said and done to people. You’re wiping the slate clean basically, you’re starting afresh” (P3)
	*Relatedness*	“Er, secondly there’s obviously the cultural value of fasting around family, around friends, particular time of year. When you’re up in the morning to close your fast you know that everybody in the street is awake” (P3) “Then there’s like the, there’s also like the social aspect of Ramadan coz like family’s come together a lot more in Ramadan. You meet a lot more friends for Iftar and stuff like that so, yeah er” (P5) “I think when you fast in Ramadan like everyone’s doing it sort of thing, it’s a group thing isn’t it really” (FgP4)
	Less talking during the fast	“I, tend to kind of reduce my, erm [pause], interactions with people, simply because of I just **talk less** during Ramadan. Erm, so that’s something that, that is impacted” (P1) “you tend to talk less because you don’t really have a lot of energy to speak to all erm the guys around the workplace. So, what I tend to do is I don’t really speak to anyone. Until or unless it’s very important and there’s something I need to speak to if not, **I don’t really speak**” (P4)
	*Competence*	‘I think for me like, they could always make up their fast afterwards. But then again, **it’s hard when you’re not doing it with everyone**, you’re doing it on your own, which is a lot worse’ (P3)
**Social** **Determinants of Food Choice**	Family *Autonomy*	‘sort of erm wary of **family** bakes that you have to eat that really coz I can’t be bothered making my own stuff” (P2) “I tend to have like a curry once a week or twice a week if my **mums** really forced it down my throat” (P3)
	Eating heavy *Relatedness* *Autonomy*	“it’s easy to actually eat, to eat worse because you’re eating a lot of fried food and you’re eating a lot of fatty foods, maybe they’re in the morning meals and then **fatty and fried foods** for the Iftar” (P1) “I just eat in the evening but then you’re eating whatever er is, is there. So it could be like erm a lot of **fried food**” (P2) “you’re starving all day and then all of a sudden you’re **eating so much**. It doesn’t make sense that doesn’t give you the sense of Ramadan either” (P4) “I didn’t eat any fried food at all but when I did go to my friend’s house, if they’ve had fried food then I would eat it” (P7)
**Engaging with Wider Society on Ramadan**	Perceived awareness and readiness among wider society to engage	“**people are quite interested** and want to know more and they’re fascinated”… “Ramadan is a fantastic opportunity to engage with people of other faiths and bring them into you know” (P1) “it’s becoming more and more, more and more erm, there’s more and **more awareness** of it in like health, from a health perspective” (P2)
	Fasting as an obligation *Autonomy*	“it’s also obligatory. Er, so as a practising Muslim, er you know this is an **obligation**, so it’s **not just a kind of voluntary thing** that you’re doing, it’s actually an obligation” (P1) “If there was a better understanding of, oh, they have to do this, they **don’t really have a choice** then they might be a little bit more accommodating” (P6)
	Working with non-Muslim colleagues	“employers may see there might be some positives in this for them, they might be more, employees might be more productive, erm so there’s actually, they could be some tangible benefits for them as well as employees” (P1) “they were changing a few things …that nurse who were fasting could have a break, to break their fast and it wasn’t very welcomed by other staff” (P7)
	Need to communicate	“Nobody’s tackled the problems, nobody has decided that we need to teach our youth what are the ways of life in modern Britain” (P3) “there’s a lot of things that going on around about Muslims and everything. So **they think we Muslims are nutters and they shouldn’t be doing this**” (P7)

### Theme 1: Experience of Ramadan for the individual

Ramadan was viewed positively as a time for personal growth. Fasting was perceived as a ‘*commitment*’ that was both physically and psychologically demanding (Table 1). Interviews consistently referred to challenges in coping with such demands through altering sleep patterns and food choices.

### Cleansing and discipline

Ramadan was viewed as a period of spiritual growth, one of *‘cleansing’ ‘detox’* and *‘renewal’*. It was also considered a time of *‘reflection’* and to exercise *‘discipline’*. Fasting allowed individuals to contemplate the *‘self’*, to review their behaviour and to become *‘healthier’* and *‘happier’*. At the same time, it was acknowledged that observing Ramadan could be demanding, both physically and psychologically and especially given the wider Western society that does not make any allowance for fasting.

*“When we live in the West erm it’s difficult to balance your deen (religion) with what the demands in the West are” (P3)*

Fasting was viewed as a commitment that required discipline.

*“It’s a discipline and then you know that when you’re fasting it’s almost like erm you’ve made a pledge and you don’t want to break that pledge” (P7)*

### Altered routine

Ramadan involves intermittent fasting, traditionally broken at iftar, after sunset, by eating dates. Participants reported disruption to their daily sleep and eating routines, including staying awake or waking up just before sunrise to eat suhoor, the pre-dawn meal, in preparation for the fast *“my routine is*, *I mean I open my fast with dates”* (P3) *“so I have my date first*, *then I’ll have my water”* (P5). Some reported not eating at all, thus extending their fasting period *“I just didn’t eat anything”* (P2).

### Attention, mood

Disrupted routines were spontaneously referred to in describing experiences of fasting. Change in routine related not only to eating, but also to sleep with references to becoming *‘nocturnal’* and having as little as *‘three hours a night’* sleep. Psychological challenges encountered when fasting were attributed to disrupted sleep and perceived to impact upon ability to function with reports of having to work more slowly and to repeat tasks.

*“I’d have to go through it like a couple of times in different ways to actually get it into my head”* (P1)

Reported negative impacts of fasting were focussed upon attention, which was considered the most affected cognitive function (Table 1), and which compelled one person to abandon the fast in order to cope with exams.

*“It leads to the point where I had to stop fasting because I could not concentrate on my exams”* (P3)

Participants also experienced tiredness and low mood, with reports of feeling irritable and angry ‘*I remember being very angry”* (P7). Through conserving energy and making healthier food choices, they were able to boost energy and manage negative impacts of fasting.

*“Well, you try to, you make healthy choices because you want to erm preserve your energy and everything”* (P7)

### Food choice and instrumental eating

Having first opened their fast with dates, the types of food selected fell into three broad categories, the major food group being fruit, while other food groups mentioned were foods referred to as *‘healthy’* and traditional *‘fried’* foods. All participants mentioned selecting fruit when opening their fast “*I eat a lot more fruit*”. While some then consumed a typical Western breakfast “*So maybe some porridge or Weetabix or*, *bananas*” (P1) or “*some bread*, *eggs*” (P7), others chose typically South Asian foods “*have like the paratha (fried chappati) in the morning*” (P5).

Iftar, the breaking of the fast at sunset, is traditionally marked with the consumption of fried South Asian foods such as *‘samosas’*,*‘kebabs’* and *‘paratha’* which were considered the traditional during Ramadan, characteristic of the food culture and symbolic of social eating. Eating *‘light’* was a major theme as was eating *‘heavy’*. In practice, food choices tended to be a blend of British and South Asian foods including traditional *‘heavier’* fried foods along with lighter *‘grilled’* food and *‘fruit’*.

“*Like I might grill a small piece of fish or something* … *which is still a light meal*” (P3)

“*Fried food I guess and erm a lot of fruit*” (P8)

Narratives implied that people were eating instrumentally to manage their fast so that they could function physically and psychologically despite the disrupted sleeping and eating routine amidst a Western society. Instrumental eating was a major sub-theme, the practice of which was informed by past experiences of fasting (Table 1). Food choices were directed towards controlling *‘sugar levels’*, avoiding *‘aches and pains’* and *‘preserving energy’*. Choosing lighter foods, for example, was framed as a way of avoiding fatigue and keeping active.

“*What I’ve found is before when I used to fast I just used to eat oily foods and crap. So as soon as you’d eat, I’d just sit and I’d feel bloated out and that I couldn’t move. So now what I’ve sort of done with my meals is I’ll have salmon and veg or grilled chicken and veg*” (P5)

There were reports of food cravings *“I get really strong cravings throughout the day”* (P6) particularly for heavier high-energy foods.

“*But during the day I’ll be like I want chocolate cake, I want a cheesecake* …. *I don’t think I crave as much on normal days as I do in Ramadan*” (P5)

Reports indicated variation in body weight change during fasting with increases body weight attributed to the disrupted routine (Table 1).

### Theme 2: Experience of Ramadan in the social context

Ramadan was experienced as a time of heightened social connectivity which brought about a sense of relatedness that was perceived to enhance motivation and the ability to fast (Table 2). Social activity, however, was also perceived as a driver of less healthy ‘*heavy*’ food choices.

### Relationships and communality

Heightened social activity was considered an opportunity to connect with like-minded people and to improve and realise the value of relationships. Ramadan afforded the individual to be part of a wider collective movement around fasting and food-related activity *“it’s a group thing*, *isn’t it”* and was associated with *‘starting afresh’* with relationships, a time for forgiveness and an opportunity to *‘wipe the slate clean’*. Although the fast is a group activity there were also reports of being quiet and *‘less talking’* while fasting (Table 2).

There was the expectation that everyone was fasting and the notion that fasting would be difficult without the social support *‘it’s hard when you’re not doing it with everyone’*. At the same time, it was emphasised that although fasting was an *‘obligation’* and that they *‘don’t really have a choice’*, it was an activity that they elected to engage with and was *‘not to be missed’*. The social context of fasting was perceived to motivate the choice to fast and increase motivation to observe the practice. The experience of relatedness, along with a sense of autonomy and socially enhanced competence, together, appeared to exert a potent influence, even among those who claimed to be less religious.

*“You know I wouldn’t say I was* *really* *religious but erm, I think erm fasting I, I just [pause] I don’t want to miss fasting so, yeah*” (P2)

### Sharing food and autonomy

Fasting is traditionally followed by Iftar, which commences at sundown and is characterised by social activity around the sharing of food. Social gatherings surrounded traditional fried foods which were considered *‘not very healthy at all really’*, but which people felt obliged to eat them when they were offered.

“*I didn’t eat any fried food at all but when I did go to my friend’s house, if they’ve had fried food then I would eat it*” (P7)

Food choices were also determined by household composition. Contrasting views reflected living arrangements, whereby those living alone or cooking own meals considered themselves to have greater autonomy of food choice and to be selecting ‘healthier’ options.

“*Because I don’t live with my parents, I don’t live with anyone, I live on my own. So a… I’ve got that advantage, b: I’m not really spending time in the kitchen I don’t really. I’m a bit lazy actually so I don’t really tend to cook. Or maybe if I do something I try to eat very healthy kind of tuna or maybe sandwiches*” (P5)

### Gender and the experience of Ramadan

There was consensus that a ‘Ramadan cook’ was a woman and some suggestion that fasting may be experienced differently by men and women and the preparation of food following Iftar was considered the domain of women.

“*Erm, I think my family, my wife and my mum in particular pay more attention to what we’re eating and a lot of my friends and you know, extended family do the same. Erm, I don’t get away with that, the wife don’t let me get away with that, so I have to go home, I have to help with the kids, I help with the cooking, go shopping whatever*” (P3)

Others considered fasting to be more difficult for women because their roles involved cooking and preparing food for the family.

*“I think it’s harder for women because if you think about it women have to make the food”* (P8)

### Theme 3. Experience of Ramadan in a Western culture and environment

There was a lot of dialogue on the challenges faced in fasting while being part of a culture that does not recognise the practice (Table 3). Subthemes were concerned with adapting, cultural exchange and the need for policies to enable flexible working and educational practices during the fast.

### Western culture and climate

As fasting occurs during daylight, in the UK it can last between 16–18 hours. A major concern was dehydration, especially when Ramadan falls in the summer. Hydration was achieved through avoidance of caffeinated beverages “*I completely ignore coffee … don’t drink any sort of tea or anything like that*” (P4) and by drinking plenty of water and fluids prior to fasting.

“*I used to drink a lot of water before I went to sleep or try get up er, erm and drink some water, drink a glass of water*” (P2)

British Ramadan food choices were considered as healthier than those in predominately Islamic countries.

“*Unfortunately in like erm Arab countries. So when I go to back home for erm for Ramadan, I see that it was the complete opposite where you’d have SO much food on the table and so many different types of like rice and like and like everything. Like people would eat a lot more in Ramadan and actually people would put on weight and be more unhealthy but, I feel like over here people are a bit more conscious about that*” (P6)

Reference was made to a *‘new generation of modern British Muslims’* who experience difficulties in observing the fast and would welcome some adjustment to work and study routines to bring them into line with the light/dark cycle of the northern hemisphere (Table 3).

### Cultural exchange

There was emphasis upon the need for communication and cultural exchange to enable Western society to accommodate and adopt to the practice of Ramadan (Table 3). There was also an awareness of the need to adapt Islamic practices to the Western environment and society and to accept British tradition and laws.

*“We live in a country where we have to recognise the laws and regulations of the country and I’m talking as a solicitor now, the laws and regulations of the country are paramount. We can’t break those laws and regulation, so our deen has to fit in. We can’t just say this is our deen and so whichever laws we want from the UK we’ll adapt. No, we have to adapt to these laws before we consider those laws. So the fact of the matter is that, we have to learn to integrate and psychologically, we have to accept where we are”* (P3)

### Impact of fasting upon work/education

Participants identified not only as Muslims, but also as citizens with professional roles in UK society, and this was reflected in an expressed need for greater communication on Ramadan practices. People reported adapting and making changes to the work routine as well as booking holiday leave to coincide with the fasting. Suggested adjustments to the workplace included the timing of breaks and greater flexible working (Table 3). There was praise where there had been communication and some adaptation within the workplace.

*“Where I’m working where I work there was erm the chaplains of other religion they all got together. They organised a lecture about erm Ramadan and everything which was really, really good”* (P7)

Participants felt that because Ramadan is becoming a more widely known tradition, non-Muslim colleagues were more aware of practices and will provide dates and water to those on night duty who were fasting.

*“Few things that the chief nurse did where I’m working like provide dates and water”* (P7)

Fasting Muslims intersected with non-fasting non-Muslim people daily where there could be some friction, for example, where breaks were timed to coincide with Iftar *‘it wasn’t very welcomed by other staff’*. There was the perception that *‘nobody’s tackled the problems’* and an imperative for *‘better understanding’* to enable greater accommodation to food and fast—related practices associated with Ramadan. On a positive note, there was a perceived opportunity to communicate with wider society on the ethos of Ramadan and awareness of a readiness to better understand practices among UK citizens *‘people are quite interested’*.

Questions about education and the potential difficulty in taking exams during Ramadan, elicited contrasting views. On the one hand, there was some reluctance, particularly among the male participants, to back changes to exam timetables to accommodate Ramadan around the light-dark cycle and fasting.

*“If Ramadan happens to fall during May, er when most, April or May, and obviously next year they’ll probably be, most of the exams will fall in Ramadan. Then, erm [pause] I, I don’t think that there’s a need to change, erm policy around that”* (P1)

Women referred the difficulty in accommodating fasting within education and unlike the men, held aspirations for future change to accommodate Ramadan in UK society and stressed the need for policies directed toward accommodating education/exams during Ramadan.

*“At the same time government does not have or maybe does not exercise sort of policies for the students during Ramadan. So, examinations, they can conduct outside Ramadan period, but they don’t do it because there’s no policies as such. So erm they could be more accommodating, but erm in my own personal opinion, you have to lobby the government”* (P5)

Themes related to the environmental context referred to difficulties faced not only in observing Ramadan in a Western society, but also in keeping the spirit of Ramadan alive throughout the year.

*“Unfortunately because of the society we live in and the environment that we live in, er it’s so, it kind of, erm people just kind of, the effect kind of fades away"* (P1)

There was a perceived readiness and optimism for greater integration of Muslims into UK society.

*“Yeah, I think, I think there’s been a lot of integration. If you come into Bradford in particular, you go into any environment you see white, black, brown, every colour of skin working together. It’s, it’s irrelevant what our background, our faith is. People are working together”* (P3)

## Discussion

This study has explored, via semi-structured interviews and focus group discussion, the perceptions and experiences of fasting during Ramadan in UK resident Muslims. As part of our aim to confirm data saturation, we included a younger focus group of British Pakistani women, aged 18–19 to explore whether similar themes would emerge from this demographic and to ensure that the theory developed applied across different age groups. While there were commonalities with older participants, such as the spiritual and communal aspects of fasting, younger participants emphasised challenges related to studying during Ramadan. This difference reflects how age and educational responsibilities may shape fasting experiences, and confirms the value of considering diverse perspectives to develop a holistic understanding of Ramadan fasting. These findings suggest that younger participants, particularly students, might benefit from interventions or policies that accommodate their unique needs during Ramadan.

The aim has been to inform the design of culturally appropriate interventions and policies to facilitate the practice of fasting in relation to the social ecological theory (SET) which has been applied previously in dietary health behaviour research [22].

As expected, given previous qualitative research in people living with diabetes through Ramadan [17–19], accounts of fasting were steeped in spirituality alongside notions of discipline, cleansing and renewal, all of which were considered important to psychological wellbeing. At the individual level, practices around fasting were perceived to alter sleep and eating patterns. According to SET this disruption to routine in order to accommodate fasting could represent a lack of congruity between the individual and the wider context in which they operate, which could have consequences for lifestyle choices, psychological function and wellbeing that require policy-level intervention.

Although existing evidence for cognitive change during Ramadan fasting is mixed with many studies indicating no cognitive deficit [4–6, 37], participants in our study unanimously referred to impaired attention. This could be because our participants, unlike those in many previous studies, were observing Ramadan in a Western culture where they had to cope with everyday life and work despite disrupted routines. This perceived cognitive deficit is in keeping with findings from previous quantitative research [2, 3] indicating a need for policies that accommodate and enable Muslims to practice fasting in Western societies.

Participants reported mood changes and increased feelings of fatigue. While evidence for mood alterations during Ramadan is mixed, our findings align with quantitative research [37], showing mood decline but contrast with studies reporting mood improvement [6–8]. Previous quantitative research should be interpreted with caution given the small samples employed, [6, 7] highlighting the need for further investigation.

Similar to existing qualitative studies of fasting during Ramadan [15, 19], participants in our study engaged in spontaneous discussion about dietary practices and healthy eating during Ramadan. A novel finding was the consistent mention of instrumental eating practices directed toward controlling blood sugar levels, combatting fatigue, enhancing cognitive function, stabilising mood and preventing stomach cramps. Participants commonly choose lighter foods, opting for more fruit and less fried food. Although some discussed weight gain and loss, unlike other qualitative research findings [15], there was little evidence of heightened anxiety around body image during fasting.

Previous qualitative studies highlight the social benefits of Ramadan fasting [14–16, 30]. Participants in our study also valued sharing food, which renewed and enhance feelings of connectedness. While existing research emphasises the importance of social support to promote eating healthy eating and dietary behaviour changes [26–28, 32]. In contrast with previous qualitative research conducted in Bradford [38, 39], some participants perceived family and social norms to govern food choices could limit their autonomy in making food choices during Ramadan. SDT assumes that social activity is highly motivating for behaviour particularly for fostering a sense of relatedness, competence and autonomy [31]. According to SDT, social influence that coexists with perceived competence and autonomy can lead to higher intrinsic motivation which has been linked to better dietary quality [32, 33]. Collective fasting was perceived to bring about a greater sense of belonging and SDT would imply that this enhanced sense of relatedness is probably an important driver of adherence to the fasting tradition. It is therefore likely that the social connectivity experienced during Ramadan enhances perceived competency to fast. SDT also acknowledges the role of autonomy in adherence to a behaviour. Participants emphasised that although fasting was an *‘obligation’* and that they *‘don’t really have a choice’*, it was an activity they chose to engage with. They expressed gratitude for traditional fried foods offered at gatherings, yet this conflicted with their desire for lighter options to manage fasting challenges. According to SDT, limited autonomy in food choices may decrease the consumption of heavier foods, underscoring the importance of relatedness and competence in adhering to fasting.

At the environmental level, a new generation of modern British Muslims was reflected in diverse perspectives on Ramadan fasting in the UK. While recognising the challenge of the wider societal culture, some took a more traditional ‘no-help needed’ attitude, others argued a case for greater flexibility within the workplace and the education system and emphasised the importance of studying for British Muslim students. These contrasting perspectives highlighted a point of tension around Ramadan and Western culture [9, 15] indicating a need for better communicate with non-Muslims on Ramadan fasting. Given that Muslims often work as part of multi-faith teams, SDT would imply that the apparent lack of congruence between Ramadan practices and the wider (Western) social and environmental context (with resultant disruption to routines and potential associated psychological deficit), indicates that the timing of practices linked to Ramadan should be considered when adapting learning and workplace schedules to better accommodate Ramadan.

### Strengths and limitations

Consistent with the SET framework that advocates the employment of diverse methodologies [21], a combination of individual interviews and a focus group discussion has been employed to capture individual and group (normative) perspectives on fasting during Ramadan. SET provides us the ability to draw upon the wider contextual, psychological processes to make clear recommendations for future policy implementations. While this study has produced rich data on a range of issues for potential intervention, one of the limitations of this study is the relatively small sample size, and renders the findings difficult to generalise to the wider UK Muslim community, even by qualitative research standards. While we were able to reach saturation with both interviewees and focus group participants, the recruitment process was challenging due to the under-researched nature of this population. The difficulty in recruitment highlights the need for further research in this area to better understand the diverse experiences within this population. Despite the small sample size, the themes identified were consistent across participants, providing a solid foundation for further investigation. Another potential limitation of our study is that all interviews were conducted during a summer Ramadan, when fasting hours are longer due to extended daylight hours. This may impact the participants’ experiences, such as feelings of fatigue, hydration challenges, and overall routine disruption. Fasting during winter months, when daylight hours are significantly shorter, could result in a very different experience, with potentially less physical strain and altered routines. However, due to the lunar nature of the Islamic calendar, Ramadan shifts across different seasons over a 33-year cycle, meaning that it takes approximately 10 years for Ramadan to move from summer to winter. Future studies could explore these seasonal differences by capturing experiences during a winter Ramadan to compare the impacts on daily routines and overall health. The findings also highlight the need for controlled quantitative research into the affective, cognitive and social aspects of intermittent fasting and how they affect functioning. There was some suggestion that experiences of fasting may be gendered and although this could be because the sample comprised more women than men, implies a need for further study of gender differences in the experience of fasting during Ramadan.

### Policy implications

Our participants perceived a readiness among the UK society to engage with Muslims on Ramadan and for cultural exchange and a need for policies to render the practice of fasting during Ramadan less difficult in the UK. Meta synthesis of qualitative studies (N = 11) of people living with diabetes during Ramadan [19] emphasised the need to increase societal understanding of Ramadan fasting. There was acknowledgement of the need for national policies on Ramadan to enable workplaces and education establishments to adapt to related religious practices. There is also a need for increased cultural competency across workplace organisations and educational institutions with a focus on shared decision making to meet the cultural needs of Muslims observing Ramadan [40]. This would help to create a more equal society in which Muslim people are accommodated during Ramadan.

According to SET, effective policy should operate at the individual, social/community and organisational/environmental domains and integrate active and passive initiatives [21]. Supporting Muslim people at the individual level could require adjustment of exam schedules during Ramadan in further and higher education. At the societal level, employment related policies to enable flexible working for practicing Muslim workers in managing their fast and coping with any associated sleep deficit and fatigue. Policies are required to ensure that Muslim employees (where possible) can take breaks at points during the day that fit with the demands of fasting and enable them to function well. Taking the environmental context into account, raising awareness and understanding of Ramadan among non-Muslims in wider society and education on what it means for Muslim people, would further support them in their fasting. Integration of Ramadan into the national social calendar and statutory public holiday entitlement would pave the way for more specific recommendations.

## Conclusion

This appears to be one of the first qualitative studies to have considered experiences of intermittent fasting associated with Ramadan in a non-clinical sample residing in a northern hemisphere-based, Western culture. Our data indicate that fasting during Ramadan is imbedded within and perceived to impact upon all aspects of everyday life. SET holds that individual wellbeing is maximised (and discomfort minimised) when there is cohesion between biological, behavioural and socio-cultural needs and the environmental context including the structures and resources available to them [21]. Responses indicated a perceived lack of congruence (points of tension) between the Muslim person observing the tradition of fasting during Ramadan and the wider UK environment. This implies a need for interventions and policies so that Muslims feel more supported during the month of Ramadan. These findings may also have implications for the design of culturally appropriate strategies for food and lifestyle interventions for preventing non-communicable disease [39]. Meanwhile, this research has identified areas at the individual, social and environmental levels that require more specific support structures and policies put in place to accommodate people who practice the UK’s second most prevalent religion.

## Supporting information

S1 File (DOCX)
