# Supplementary material for: Experiences of fasting during Ramadan in British Muslims: Psychological, social and health behaviours
Source: PLoS One. 2025 Jan 9;20(1):e0313688. doi: 10.1371/journal.pone.0313688 (PMC11717292; doi:10.1371/journal.pone.0313688)
Supplement: S1 File — (DOCX) [file pone.0313688.s001.docx]

**Experiences of Fasting during Ramadan in British South Asians: Psychological, Social and Health Behaviour**

**Appendix 1: Interview Topic Guide**

Q. Briefly describe what Ramadan means to you?

Q. Why do you fast during Ramadan?

Q. Can you describe a typical day for you during the month of Ramadan?

Q. During Ramadan, do you think you eat more or less than a normal non-Ramadan day?

What do you usually eat and drink in the morning when you close your fast? (Sehri)

How do you usually break your fast? (Iftari)

Q. What do you do in the time-period between opening (Iftari) and closing (Sehri) your fast?

Q. Do you sleep more or less in Ramadan?

Approximately how many hours of sleep do you get?

Do you nap during the day?

Q. Does the amount of physical activity you do change?

If so, how?

Q. Does Ramadan bring about a change in your body weight?

Q. Whilst fasting, do you notice any changes in your memory or concentration?

Q. Whilst fasting, do you notice any changes in your mood?

Q. How do you manage work/studying during Ramadan?

Q. Does your work/study performance change during Ramadan?

Q. How supportive and/or accommodating is your workplace/University during Ramadan?

Q. What changes (if any) would you like to see changes in workplace/University?

Q. How accepting do you think the UK community is of fasting Muslims?

Has this changed over the years?

How can we change this (if not)?

**Appendix 2 – Focus Group Topic List/Schedule**

Q. Briefly describe what Ramadan means to you?

Q. Why do you fast during Ramadan?

Q. Can you describe a typical day for you during the month of Ramadan?

Q. Describe your daily routine during Ramadan (compared to a normal day)?

If/when sleep mentioned:

Q. How do you sleep during Ramadan?

If/when food and eating mentioned:

Q. What would you eat in the morning to close your fast?

Q. What would you typically eat to break your fast?

Q. Do you think you have a more healthy or less healthy diet in Ramadan (compared to a normal day)?

Psychological Function:

Q. How was your mood during Ramadan?

Q. How did you all cope with fasting and doing your exams at the same time?

Q. When you had exams during Ramadan, did fasting affect how you performed?

If/when perceived deficit mentioned:

Q. How did you overcome that?

Q. Was there anything that you kind of tried and tested to help you or?

Q. Do you think it would have been beneficial for you to have done your exams when you weren't fasting?

**Scenario:** Say its Ramadan during the wintertime, and there's a taxi driver and he normally picks children up from a busy primary school at 3 o'clock. So how aware and attentive do you think he would be during his job?

Q. What could/should the government do?
